# Effect of Ag loading position on the photocatalytic performance of TiO_2_ nanocolumn arrays

**DOI:** 10.3762/bjnano.11.59

**Published:** 2020-05-05

**Authors:** Jinghan Xu, Yanqi Liu, Yan Zhao

**Affiliations:** 1 Beijing Engineering Research Center of Laser Technology, Research Institute of Laser Engineering, Beijing University of Technology, No. 100 Pingle Park, Chaoyang District, Beijing 100124, People’s Republic of China; 2 Key Laboratory of Trans-scale Laser Manufacturing Technology, Ministry of Education, Research Institute of Laser Engineering, Beijing University of Technology, No. 100 Pingle Park, Chaoyang District, Beijing 100124, People’s Republic of China

**Keywords:** anodic aluminum oxide template, nanocolumn arrays, photocatalysis, surface plasmon resonance

## Abstract

Plasmonic metal/semiconductor composites have attracted great attention for efficient solar energy harvesting in photovoltaic and photocatalytic applications owing to their extremely high visible-light absorption and tuned effective band gap. In this work, Ag-loaded TiO_2_ nanocolumn (Ag-TNC) arrays were fabricated based on anodic aluminum oxide (AAO) template by combining atomic layer deposition (ALD) and vacuum evaporation. The effects of the Ag loading position and deposition thickness, and the morphology, structure and composition of Ag-deposited TNC arrays on its optical and photocatalytic properties were studied. The Ag-filled TiO_2_ (AFT) nanocolumn arrays exhibited higher removal efficiency of methylene blue (MB) compared with Ag-coated TiO_2_ (ACT) nanocolumn arrays and pure TiO_2_ nanocolumns arrays. Both experimental and theoretical simulation results demonstrated that the enhanced photocatalytic performance of AFT nanocolumn arrays was attributed to the surface plasmon resonance (SPR) of Ag and the absorption of light by TiO_2_. These results represent a promising step forward to the development of high-performance photocatalysts for energy conversion and storage.

## Introduction

Since 1972, when Shimada and Honda discovered the photocatalysis of titanium dioxide (TiO_2_) under ultraviolet light, research in this field has continued to grow [1]. Recently, TiO_2_ has been utilized in the fields of photocatalytic water decomposition [2], photocatalytic organic degradation [3], and artificial photosynthesis [4]. In particular, research on the application of TiO_2_ in the field of water pollution degradation has experienced a notable increase due to its nontoxicity, nonpollutant nature, high chemical stability, and low cost [5]. However, the absorption spectrum of pure TiO_2_ is too narrow (200–400 nm), and the high hole–electron pair recombination rate restricts its photocatalytic efficiency [6–7].

Many efforts have been devoted to solve these problems with the aim of improving the catalytic performance of TiO_2_, which include doping with nonmetal elements [8], dye sensitization [9], and combination with metal elements or other metal oxides [10]. Compared with the bulk material, one-dimensional (1D) nanostructured TiO_2_ presents enhanced photocatalytic activity that depends on a variety of factors such as surface area, particle shape, crystalline structure, crystal size, and surface active sites [11–12]. In addition, TiO_2_ combined with noble metal (e.g., Au, Ag, Pt), nanostructured to form a metal–oxide structure, has been shown to greatly improve the catalytic efficiency of the material [13]. When excited by light, noble metal nanoparticles can exhibit surface plasmon resonance (SPR), which leads to strongly absorbed visible light and enhancement of local electromagnetic fields [14]. Among the noble metals, Ag nanostructures have been widely used as catalysts because of their reasonable cost and broad plasmon resonance in the visible region [15–16]. At present, the preparation of 1D TiO_2_ nanostructures is mainly performed by using anodization [17], hydrothermal [18] and template [19] methods. Meanwhile, the most common methods for the combination with precious metals are chemical deposition [20] and physical deposition [21].

The development of anodized aluminum oxide template (AAO) has constituted an important advance in the field of film preparation, since it allows the formation of nanostructured films with a high degree of morphology control [22]. Furthermore, according to Das et al. [23], the use of atomic layer deposition (ALD) for the preparation of TiO_2_ films affords specimens with different electron transfer characteristics than those of rutile and anatase TiO_2_, and the as-prepared films fit the template well. When Ag particles are combined with TiO_2_, the photocatalytic performance of the film can be significantly enhanced by hot electron injection [24], localized surface plasmon resonance (LSPR) [25], and plasma excitation light scattering [26–27]. Cushing et al. [28] used the time-resolved method to track these three processes by establishing a density matrix model, which allowed them to predict the theoretical maximum catalytic efficiency and theoretical optimal structure of the plasma metal–semiconductor heterojunction. Having this background in mind, we decided to prepare a TiO_2_ nanocolumn (TNC) structure by using AAO in combination with ALD, in which Ag particles could be selectively supported separately outside and inside the nanocolumns.

Most of the 1D TiO_2_–Ag nanostructures prepared to date are Ag-nanoparticle-coated TiO_2_ (ACT), in which the catalytic efficiency of the catalyst is greatly decreased when the Ag loading is increased to a certain extent. Wang et al. [29] used the hydrothermal method to prepare an array of TiO_2_ nanocolumns whose outside surface was then coated with Ag particles by chemical deposition. After seven deposition cycles, the catalytic efficiency of the as-prepared TiO_2_–Ag film decreased significantly. A similar decline in the catalytic efficiency was observed in a TiO_2_ nanocolumn array fabricated by photolithography and the template method by Sung et al. [30]. In this case, Ag particles were loaded on the outside of the nanocolumns by magnetron sputtering, and the catalysis was carried out at a sputtering time of 30 min. Besides, Jani et al. [31] studied the preparation of TiO_2_ nanotube arrays by anodization, and Ag particles were loaded on the inside and the outside of the nanotubes by chemical deposition. The catalysis of this composite was carried out at a concentration of 1100 mM in AgNO_3_ solution. It was found that the loading position of precious metal particles in the TiO_2_ structure could not be regulated, which had a negative effect on the catalytic efficiency. For these reasons, we considered it worthwhile to study the effect that the position of the Ag loading may have on the catalytic efficiency of the newly prepared Ag-loaded TiO_2_ nanocolumn (Ag-TNC) arrays.

## Experimental

Aluminum sheets, chromic acid (H_2_CrO_4_), and methylene blue (MB) were purchased from Alfa Aesar. Alcohol, perchloric acid (HClO_4_), and sulfuric acid (H_2_SO_4_) were purchased from Beijing Chemical Works. All the chemicals used in the experiments were of analytical grade and used without further purification.

### Method

**Anodized aluminum template preparation:** Highly pure aluminum sheets (purity >99.9%, 20 × 20 × 0.5 mm) were subjected to secondary anodization to prepare highly ordered AAO templates. Thus, each aluminum sheet was annealed prior to oxidation and then electrochemically polished using a 1:4 volume ratio of HClO_4_ and alcohol. The anodized electrolyte solution was 0.2 M H_2_SO_4_, the oxidation voltage was 20 V, the ambient temperature was maintained at 0 °C, and the oxidation time was 6 h. After the oxidation was completed, the aluminum sheet was immersed in 1.8 wt % H_2_CrO_4_ to remove the first oxide layer. The sample was then subjected to a second oxidation treatment under the same conditions as the first oxidation step. After an oxidation time of 60 s, a single-pass AAO template with a pore diameter of 40 nm, a pore pitch of 65 nm, and a pore depth of 150 nm was obtained.

**Ag-TNC array preparation:** An array of TiO_2_ nanocolumns was deposited (300 cycles) on the AAO template using ALD (home-built) with TiCl_4_ as the precursor.

The preparation of Ag-filled TiO_2_ nanocolumns (AFT) was as follows: A sample deposited with TiO_2_ was placed in a vacuum evaporation apparatus (Shen Yang, LN-1004A) and subjected to Ag deposition at a deposition pressure of 10^−4^ Pa, an evaporation source current of 44 A, and an evaporation rate of 0.08 nm/s. The sample was then transferred onto a crystal adhesive, and the AAO was removed by immersing the sample in 0.2 M NaOH solution for 2 h to obtain the Ag–TiO_2_ array.

For the preparation process of Ag-coated TiO_2_ nanocolumns (ACT), the Ag deposition was performed after removal of the AAO template as follows. The sample deposited with TiO_2_ was supported onto crystal adhesive and then immersed in 0.2 M NaOH solution to remove residual Al and AAO. Then, the sample was cleaned, dried, and placed in a vacuum evaporation apparatus for deposition of Ag particles under the same conditions described above. We made samples with different parameters by changing the deposition time of Ag. Samples of all parameters are shown in Table 1.

**Table 1:** Summary of the preparation conditions of Ag-loaded TiO_2_ samples.

Sample code	Scheme name	Deposition time (Ag)	Deposition thickness (Ag)

TNC	TiO_2_ NC	0	0

AFT1	Ag-filled TiO_2_ NC	250 s	10 nm
AFT2	Ag-filled TiO_2_ NC	500 s	20 nm
AFT3	Ag-filled TiO_2_ NC	1000 s	40 nm
ACT1	Ag-coated TiO_2_ NC	250 s	10 nm
ACT2	Ag-coated TiO_2_ NC	500 s	20 nm
ACT3	Ag-coated TiO_2_ NC	1000 s	40 nm

### Characterization

The surface and cross-sectional morphology of the Ag-TNC structures were observed using a field emission scanning electron microscope (FE-SEM, FEI, Tecnai G2 F30). The elemental composition and valence distribution of the film were measured by X-ray photoelectron spectroscopy (XPS, Thermo Fisher Scientific, ESCALAB 250Xi). The photoluminescence (PL) intensity of the array was measured using a laser of 325 nm excitation wavelength and a grating spectrometer (Horiba, JY iHR550). The diffuse reflectance absorption spectrum of the array was measured using an ultraviolet–visible spectrophotometer (UV–vis, Shimadzu, UV-3600).

**Photocatalytic experiments.** The photocatalysis was performed under 300 W Xe light using 15 mL of a 5 ppm MB aqueous solution. Before starting the photocatalysis, the sample was immersed in the MB solution and allowed to remain in the dark for 30 min to reach the adsorption–desorption balance. Then, the solution was placed under a light source to start the photocatalysis. Every 10 min, an aliquot of 2 mL was transferred into a UV–vis cuvette, and the change in absorbance was measured using a UV–vis spectrophotometer (Shimadzu, UV-3600). After the measurement was completed, the solution was poured back into the beaker to keep the total volume unchanged.

## Results and Discussion

**Figure 1:**
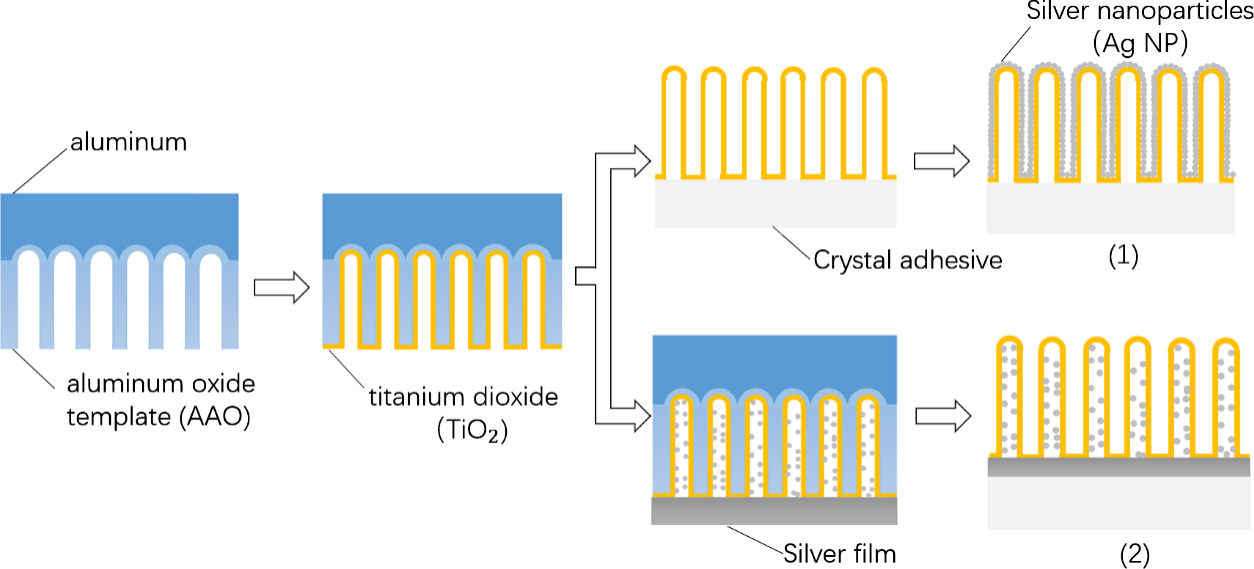
Schematic diagram of the preparation process of the different Ag–TiO_2_ nanocolumn structures. (1) Structure of samples ACT1, ACT2, and ACT3; (2) structure of samples AFT1, AFT2, and AFT3.

Figure 1 depicts a schematic diagram of the preparation process of the ACT and AFT nanocolumn arrays (schemes (1) and (2), respectively). In the structure represented in (2), the bottom of the array hinders the dense accumulation of Ag nanoparticles in the nanocolums or the formation of agglomerates. Instead, the Ag-filled TiO_2_ nanocolumn structure (2) is obtained. On the other hand, the Ag-coated structure (1) is formed without the hindrance of the bottom structure, in which the Ag nanoparticles deposit more densely and form larger nanoparticles. The structural differences between the ACT and AFT arrays depicted in (1) and (2) can be invoked as the main reason for the differences observed in their physical and catalytic properties, which will be discussed later.

**Figure 2:**
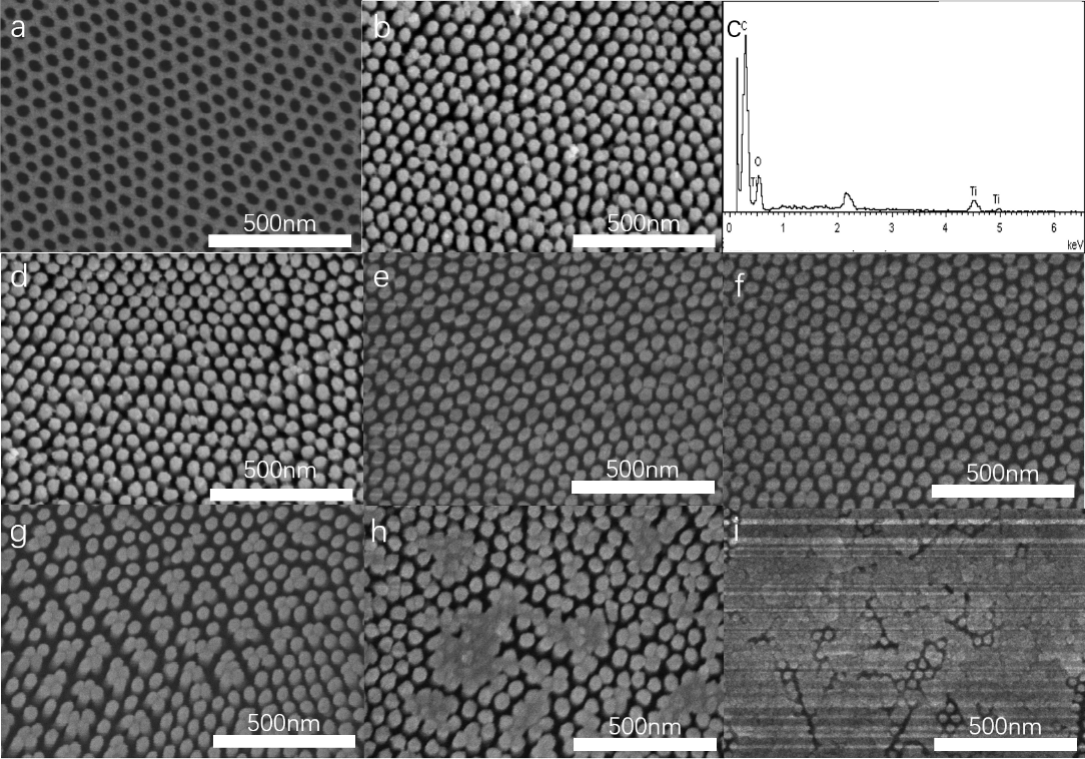
SEM images of the top view of different samples: (a) AAO template, (b) TNC, (c) EDS of TNC, (d) AFT1, (e) AFT2, (f) AFT3, (g) ACT1, (h) ACT2, and (i) ACT3.

Figure 2 shows SEM images of the top view of all samples and the elemental composition of TNC, from which the effects of different Ag deposition thicknesses and cladding structures can be observed. Figure 2a and 2b shows the morphology of the prepared AAO template and the TiO_2_ nanocolumn array prior to the Ag deposition, respectively. As can be seen, ALD perfectly replicates the template structure. Figure 2c shows the energy-dispersive X-ray spectroscopy (EDS) spectrum of TNC, and we find that the elements of the array are mainly composed of C, O, and Ti. Among them, C comes from organic substrates. Figure 2d, 2e, and 2f are AFT structures with thickness of Ag deposition of 10, 20, and 40 nm, respectively. From the observations from these figures, it can be concluded that the deposition of Ag does not affect the structure of TiO_2_, and the array of nanocolumns is well preserved and consistent with the deposition. Meanwhile, Figure 2g, 2h, and 2i show the ACT structures, which exhibit Ag deposition thicknesses of 10, 20, and 40 nm, respectively. We can see that, as the thickness of Ag deposition increases, the array of TiO_2_ is gradually covered until the nanocolumn structure disappears.

**Figure 3:**
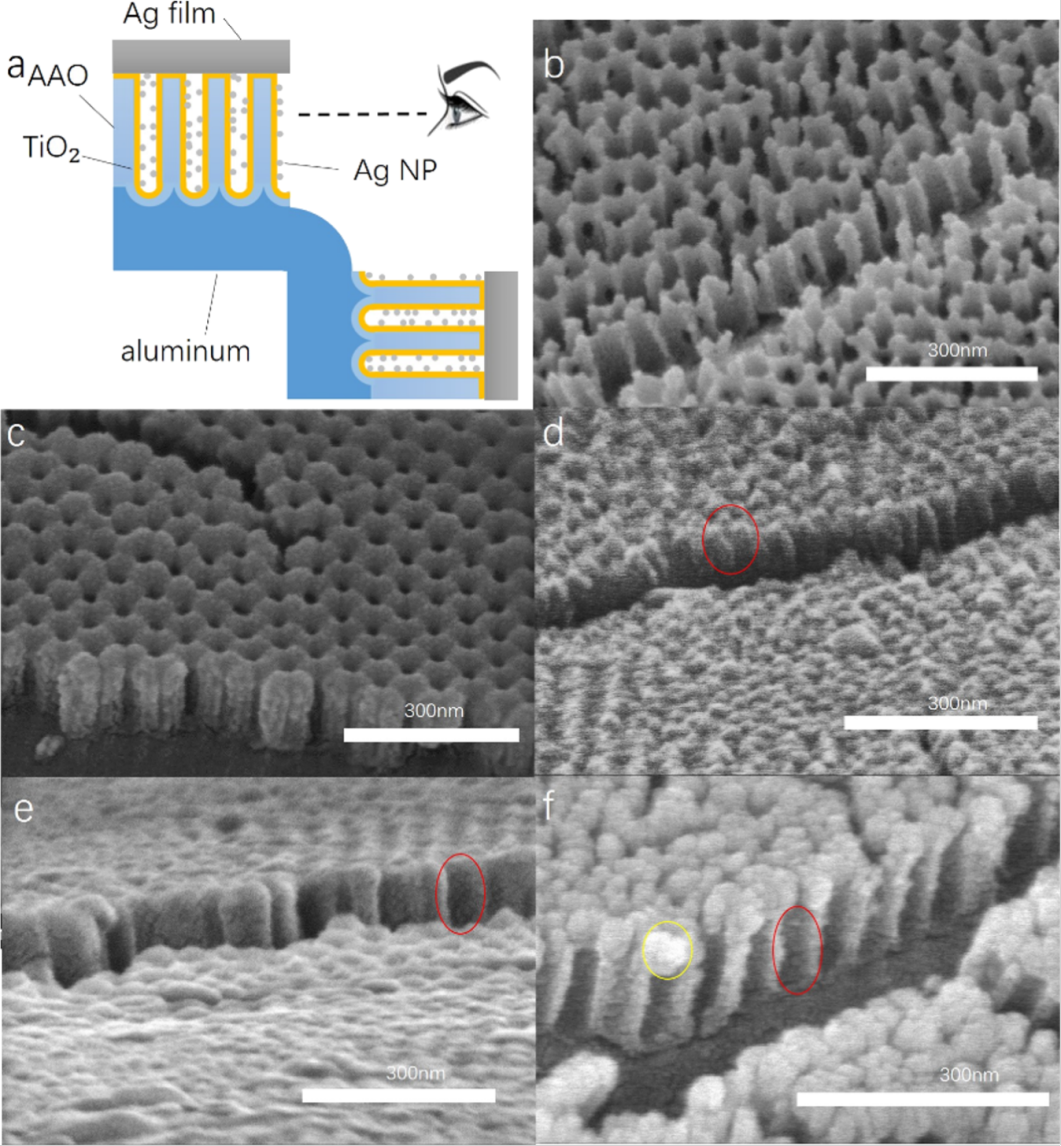
SEM cross section of samples with AAO and Al substrate. (a) Observation sketch, (b) AAO template, (c) TNC, (d) AFT1, (e) AFT2, and (f) AFT3.

To observe the distribution of Ag nanoparticles in the AFT nanocolumn array, the AAO template was broken by bending the samples prior to removal of Al and AAO, exposing the internal structure of the nanocolumn as shown in Figure 3. Figure 3a shows the method used to observe the cross section of the sample. Figure 3b is the cross section of the AAO template, and we find that there is no coverage of the surface of the template. Figure 3c shows the cross section of a TNC sample. Comparing Figure 3b and 3c, we find that TiO_2_ perfectly covers the surface of AAO, the nanocolumn is a hollow structure, and the top hole still exists. As can be seen from Figure 3d, due to the low thickness of Ag deposition in AFT1, no obvious nanoparticles can be observed in the TNC array, and not much Ag aggregation is present around the nanopore marked with the red circle. This implies that Ag can still enter the nanocolumn easily. In contrast, Ag nanoparticles can be clearly seen on the inner wall of the TNC of AFT2 marked with the red circle in Figure 3e. From the comparison of Figure 3d and 3e, it can be concluded that the Ag particles deposited in the TNC become gradually larger, but no accumulation around the nanopores occurs, which would block further deposition of Ag particles. Finally, in the structure having the thickest layer of Ag (AFT3), a large amount of Ag accumulation can be observed around the nanopore marked with the yellow circle in Figure 3f, which closes the nanopore and hinders excessive Ag deposition in the TNC. Comparing the areas marked with the red circles in Figure 3e and 3f, it becomes apparent that the increase of Ag deposition thickness does not significantly increase the size and distribution density of the nanoparticles in the TNC, and that the Ag nanoparticles still exhibit appropriate size and good dispersion.

**Figure 4:**
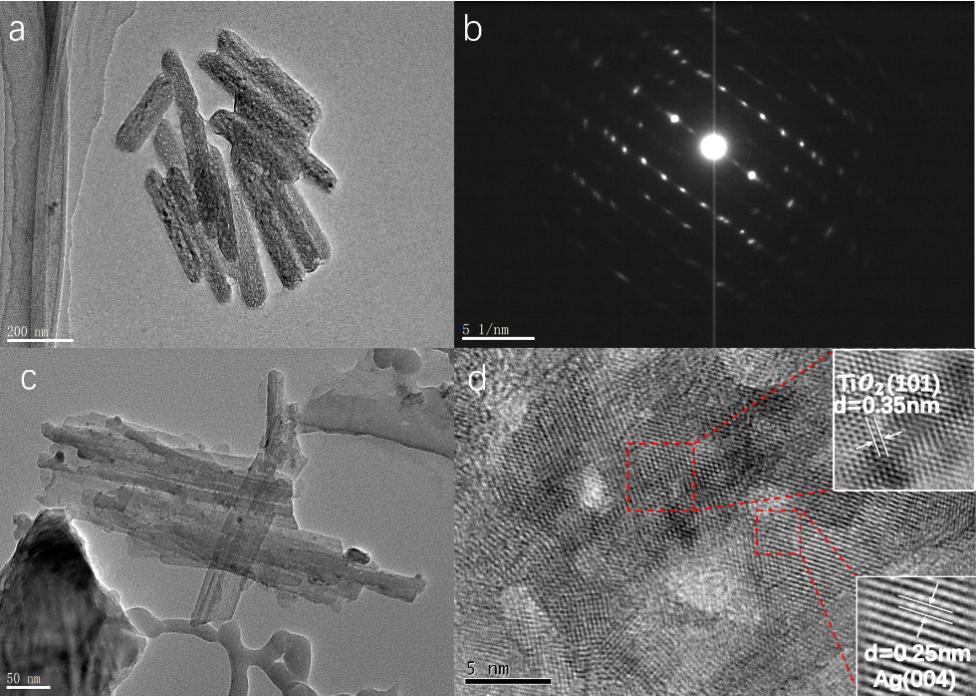
TEM images of nanocolumn structures. a) TNC nanocolumn TEM image, b) TNC TEM diffraction pattern, c) AFT1 TEM image, d) AFT1 nanocolumn high-resolution TEM image, where the insets show the image processed by the software in the selected areas of the red-dotted line frames.

The fine structure of nanocolumns and the distribution of Ag particles inside the nanocolumn array were observed by TEM. We also studied the crystal structure of the TiO_2_ nanocolumns. Figure 4 shows the TEM and high-resolution spectra of the nanocolumn structure. Figure 4a shows the bare TiO_2_ nanocolumns without Ag. It can be seen from the figure that the size of the nanocolumns conforms to the structural parameters of the AAO template. Figure 4b shows the diffraction pattern of TiO_2_ nanocolumns. According to diffraction spot analysis, it can be found that the prepared TiO_2_ has a polycrystalline structure. Figure 4c is a TEM image of the nanopillars of the AFT structure. It can be seen from the figure that tiny Ag nanoparticles are supported on the TiO_2_ columns. Figure 4d shows the high-resolution TEM image of the Ag nanocolumns shown in Figure 4c. After removing the noise by performing Fourier transform on the selected area using software, we can clearly see lattice fringes in the material from the illustration. Comparing the with the PDF card, it can be found that the fringe spacing in the upper right illustration is 0.35 nm, which corresponds to the (101) crystal plane of the anatase structure TiO_2_, and the fringe spacing in the bottom right illustration is 0.25 nm, which corresponds to the (004) crystal of Ag surface. From the comprehensive TEM results, the silver-loaded TiO_2_ array is composed of Ag particles and TiO_2_ with anatase configuration.

**Figure 5:**
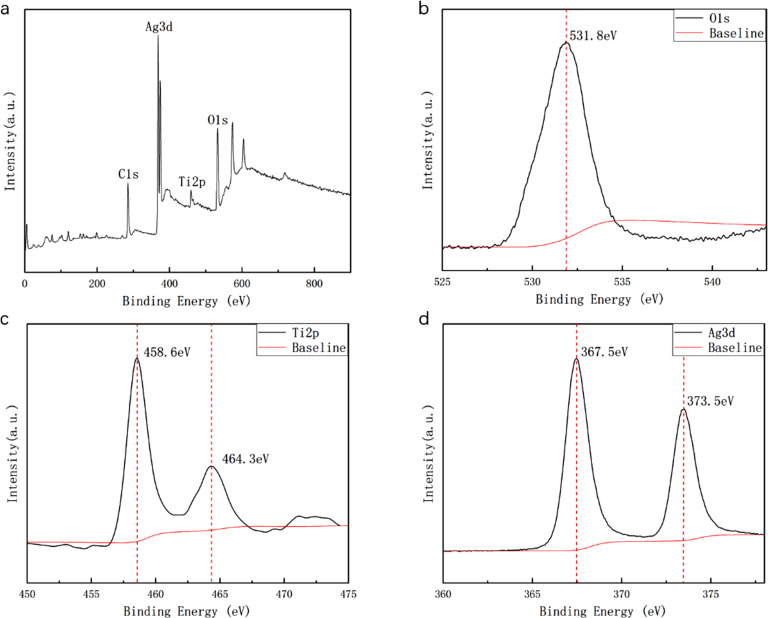
XPS spectrum of AFT3: (a) Survey spectrum, and details of the regions related to (b) O 1s, (c) Ti 2p, (d) Ag 3d.

Next, sample AFT3 was subjected to XPS analysis to characterize the elemental composition and chemical state of the Ag-TNC film, and the result is shown in Figure 5. According to Figure 5a, all the peaks can be attributed to Ti, O, C, and Ag, which indicates that the sample consists of TiO_2_ and Ag. The appearance of the C element is attributable to contamination from the cavity of the XPS device and/or from the adhesive substrate. The high-resolution spectra of Ag, Ti, and O are shown in Figure 5b, 5c, and 5d, respectively. In the spectrum of O 1s (Figure 5b), the characteristic peak is observed at 531.8 eV. The shape of this peak is symmetrical, which is indicative of the presence of one distinct O species in the sample. Meanwhile, the two characteristic peaks of Ti 2p are located at 458.6 and 464.3 eV in Figure 5c. These peaks represent the binding energy of Ti 2p_3/2_ and Ti 2p_1/2_, which indicates that Ti is in the +4 valence state in the sample. The spacing between the two peaks of 5.7 eV is also consistent with the +4 oxidation state [32]. Figure 5d shows the high-resolution spectrum of Ag. As can be seen from the figure, Ag 3d has two characteristic peaks at 367.5 and 373.5 eV, which are attributed to the binding energy of Ag 3d_5/2_ and Ag 3d_3/2_, with a spacing of 6 eV. This indicates that the structure of Ag in the sample is simple. Therefore, the results of the XPS analysis confirm that the sample is composed of TiO_2_ and Ag.

**Figure 6:**
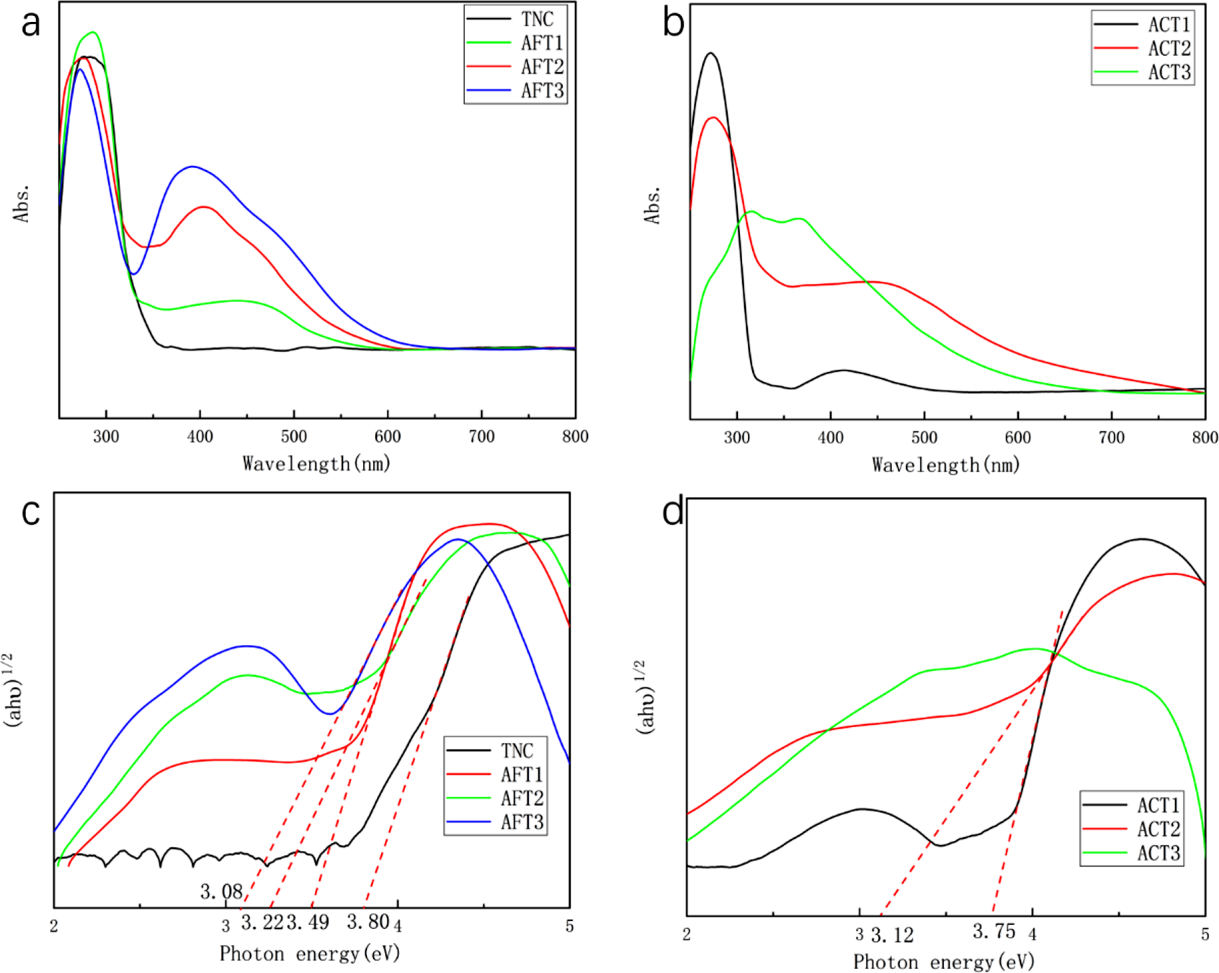
(a,b) UV–vis absorption spectra and (c,d) bandgaps of the different samples.

In order to investigate the absorption properties of the different arrays, the optical absorption tests of all samples were carried out, as shown in Figure 6. Figure 6a is the optical absorption spectra of TNC and AFT structure samples. It can be seen that the absorption peak of TiO_2_ is located at 276 nm and the absorption of the samples in the visible light portion increase as the thickness of Ag increases. Moreover, the increase of the Ag thickness does not hinder the absorption of TiO_2_ in the ultraviolet region. Figure 6b is the optical absorption spectra of ACT structure samples. It can be seen that, with the increase of Ag deposition, the visible absorption intensity of the samples increases gradually, but the absorption peak of TiO_2_ at 276 nm decreases obviously. The absorption peak of ACT3 at 317 nm is the characteristic absorption peak of Ag prepared by vacuum evaporation, and the absorption peak at 364 nm is generated by the LSPR [33].

Figure 6c and 6d show the Tauc plots of the UV–vis spectra depicted in Figure 6a and 6b, which are calculated using Equation 1 for an indirect bandgap semiconductor, since the grains are small and the energy levels are discrete:

[1] $${\left(\alpha h\nu \right)}^{\frac{1}{2}}=A\left(h\nu -E_{\text{g}}\right).$$

In Equation 1, *h* is Plank’s constant (6.626 × 10^−34^ J s), ν is the frequency of light, and *E*_g_ represents the bandgap energy. For the calculation of *E*_g_ by the Tauc plot absorbance the value of the absorption (Abs. in Equation 2) can be used directly instead of α; therefore, the *E*_g_ numerical value is determined by the intersection of the extension line and the *x*-axis (*h*ν) [34]. It can be seen from Figure 6c that the TiO_2_ specimens prepared by ALD exhibit higher *E*_g_ (3.8 eV) than that obtained using the conventional method (3.20 eV). This is attributed to the quantum size effect, according to which the *E*_g_ value of a semiconductor depends on physicochemical properties such as size, surface area, and crystalline phase [35]. When Ag particles are combined with TiO_2_, the band gap of the resulting Ag-TNC film is narrowed under the action of LSPR and hot electron injection, and the samples have absorption peaks in the visible part. In Figure 6d, since Ag covers TiO_2_, the light absorption of ACT3 is substantially generated by Ag, so the sample has no bandgap.

**Figure 7:**
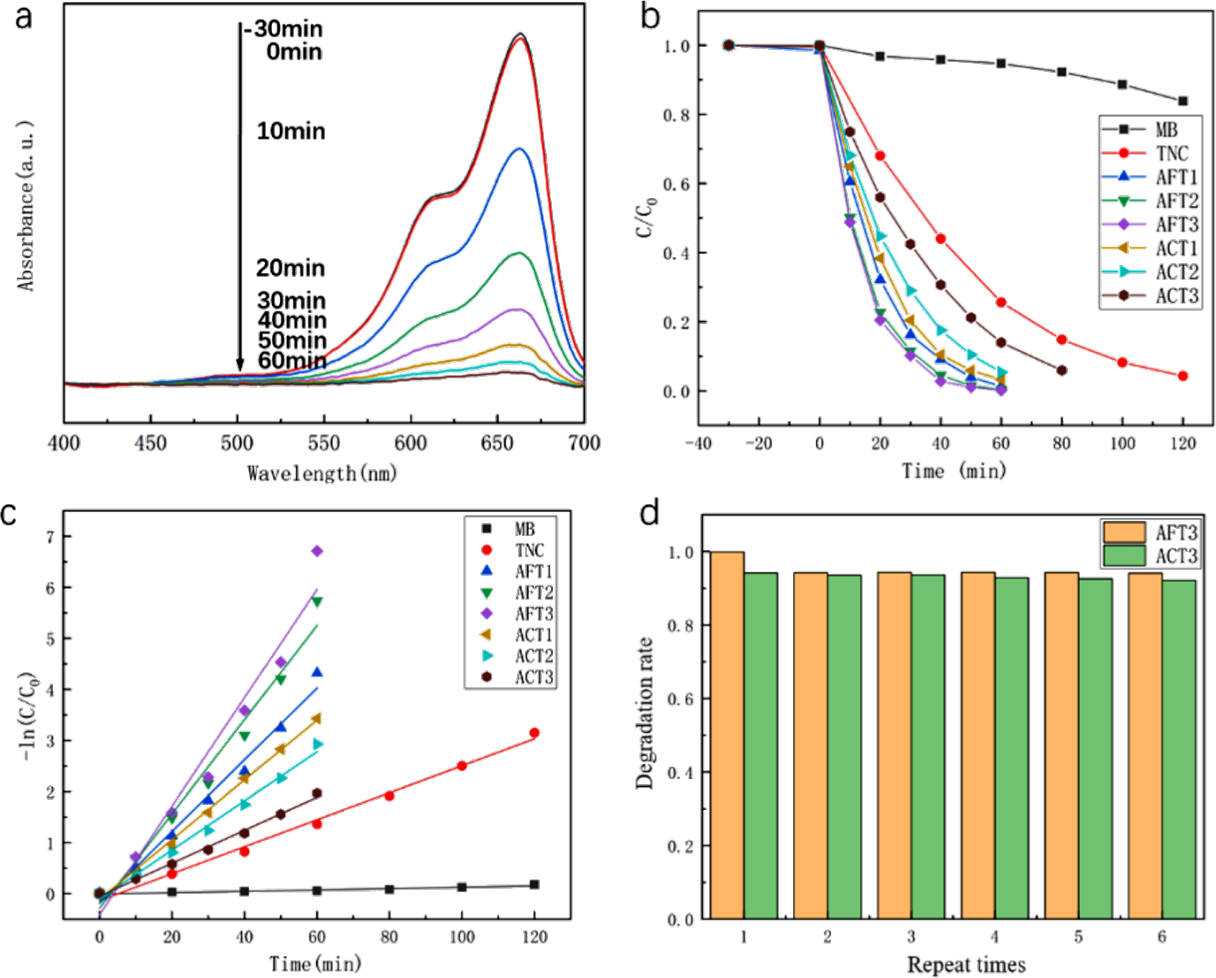
Photocatalytic activity for the degradation of MB: (a) UV–vis absorption spectra for the photocatalytic degradation of MB in the presence of AFT1 sample, (b) photocatalytic degradation rate of MB by different samples and pure MB under UV–vis light, (c) kinetics of different samples, (d) recycling of AFT3 and ACT3.

The photocatalytic reaction takes place on the surface of the catalyst, so the catalyst needs to have a certain adsorption capacity for dye molecules. Due to the presence of oxygen vacancies, the surface of TiO_2_ is usually negatively charged and has a good adsorption capacity for cationic dye molecules [36]. Commonly used cationic dyes are rhodamine, methyl blue, methylene blue, etc. This article uses methylene blue (MB) as the dye molecule. To evaluate the influence of the different structures on the catalytic reaction, UV–vis photocatalytic experiments were carried out on different samples using MB solution as the reactant, as shown in Figure 7. In these experiments, the degradation efficiency can be described by the Lambert–Beer law and the Langmuir–Hinshelwood model as follows:

[2] Abs.=abC

[3] $$\eta =\frac{C_{0}-C}{C_{0}}\times 100\%$$

[4] $$\ln\left(\frac{C}{C_{0}}\right)=-K_{\text{α}}t$$

where Abs. is the solution absorbance, *a* is the solution absorbance coefficient, *b* is the optical path, *C* is the solution concentration, *C*_0_ is the initial concentration, and *K*_α_ is the apparent ﬁrst-order rate constant.

Figure 7a shows that the AFT1 sample catalyzes the degradation of MB at different times. After 60 min of irradiation, the absorption intensity of MB at 663 nm substantially disappears, indicating that almost all the MB molecules are catalytically decomposed. Figure 7c shows the degradation efficiency of different samples under Xe lamp illumination. It can be seen that the combination of Ag and TiO_2_ results in a great improvement of the MB degradation efficiency. The Ag–TiO_2_ specimens can achieve a decomposition rate of 96.9% after 60 min illumination, whereas TNC only reaches a decomposition rate of 71.4%. This is because the absorption spectrum of the sample is broadened in the presence of Ag particles. Under the same illumination conditions, more photogenerated carriers are generated in the Ag-TNC sample, and the transfer of the photogenerated electrons to the Ag particles reduces the recombination of electrons and holes, thereby increasing the number of electrons and holes involved in the catalytic reaction. From the comparison of the Ag-loaded structures, it can be seen that AFT1 and ACT1 have very close catalytic efficiencies, indicating that the loading position does not affect the catalytic efficiency of the sample at low deposition thickness. In contrast, when the Ag deposition thickness increases, the catalytic efficiency of AFT remains unaltered, whereas that of ACT undergoes a significant decrease. This decrease is further evidenced in Figure 7b, in which the *K*_α_ from Equation 3 of samples ACT1, ACT2, and ACT3 gradually decreases, and specific *K*_α_ values are shown in Table 2. Figure 7d shows the stability of samples ACT3 and AFT3, where it can be seen that, after repeating the photocatalytic process five times, both samples maintain an efficiency close to the first catalytic cycle. This result indicates that the samples prepared by the combination of ALD and vacuum evaporation have good stability, which is not affected by the different loading structures, i.e., Ag-coated or Ag-filled.

**Table 2:** Summary of kinetic constants of thebphotocatalytic reaction.

Sample code	Kinetic constants of chemical reactions (*K*_α_)

TNC	0.0264
AFT1	0.0702
AFT2	0.0923
AFT3	0.1064
ACT1	0.0685
ACT2	0.0481
ACT3	0.0322

**Figure 8:**
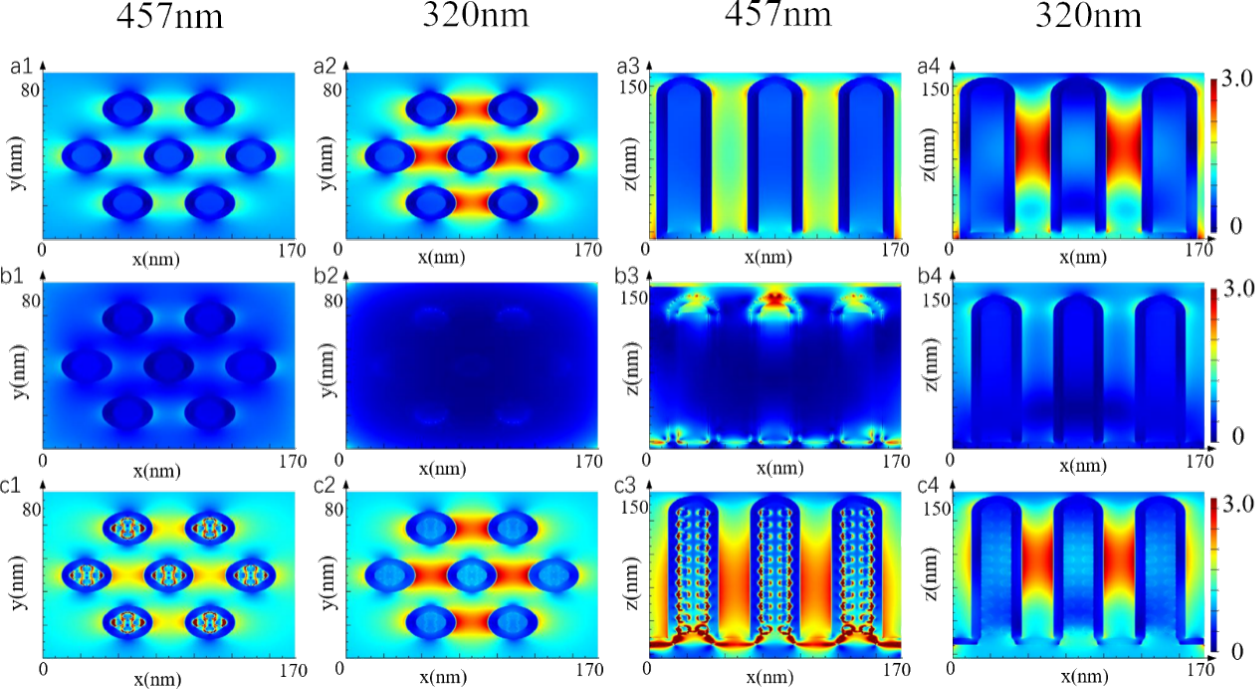
FDTD simulations of the electric field distribution of Ag-loaded TiO_2_ nanocolumns with different structures. a1) Wavelength 457 nm TNC cross section, a2) wavelength 320 nm TNC cross section, a3) wavelength 457 nm TNC longitudinal section, a4) wavelength 320 nm TNC longitudinal section, b1) wavelength 457 nm ACT3 cross section, b2) wavelength 320 nm ACT3 cross section, b3) wavelength 457 nm ACT3 longitudinal section, b4) wavelength 320 nm ACT3 longitudinal section, c1) wavelength 457 nm AFT3 cross section, c2) wavelength 320 nm AFT3 cross section, c3) wavelength 457 nm AFT3 longitudinal section, c4) wavelength 320 nm AFT3 longitudinal section.

In order to elucidate the mechanism of the influence of Ag loading position and deposition thickness on the photocatalytic reaction, we used a finite-difference time-domain (FDTD) simulation to study the electromagnetic field distribution of Ag-loaded TiO_2_ arrays with different structures. Figure 8 shows the simulation of the electric field distribution of arrays with different structures at 457 and 320 nm. In order to further illustrate the effect of structural changes on the electric field distribution of arrays, the XY plane and the XZ plane are chosen to analyze the arrays. The XY plane chooses the cross section at 75 nm and the XZ plane chooses the cross section at 0 nm. The structure of the model was established according to the morphology observed by SEM. As can be seen from Figure 8 (a1–4), the electric field of the TiO_2_ arrays is stronger under 320 nm illumination, and there is a strong electric field distribution between the nanocolumns at the height of 50–120 nm. Figure 8 (b1–4) shows results from the simulations of the ACT3 structure. Ag was filled between the nanocolumns, and a layer of 15 nm thick Ag film was covered on the top of the array. It can be seen from the figure that the Ag coating hinders the absorption of ultraviolet light by TiO_2_, and the electric field intensity between nanocolumns decreases obviously. Only under 457 nm light can a strong electric field distribution at the top of the array be observed, which is attributed to the absorption of visible light by the LSPR resonance of Ag. Figure 8 (c1–4) shows the results of the simulation of the AFT3 structure. The diameter of Ag particles in the nanocolumn is 8 nm, the distance between the horizontal and vertical spherical centers is 11 nm, the distance between the vertical spherical centers is 14 nm, and the diameter of Ag particles at the bottom of the nanocolumn is 15 nm. At the same time, the bottom of the nanocolumn is covered with a Ag film of 15 nm thickness. It can be seen from the figure that under 457 nm illumination, due to the LSPR effect of Ag particles, a strong electric field is generated on the Ag particles, and hot electrons are injected intoTiO_2_, so the electric field intensity at the interface between Ag and TiO_2_ is the highest.

**Figure 9:**
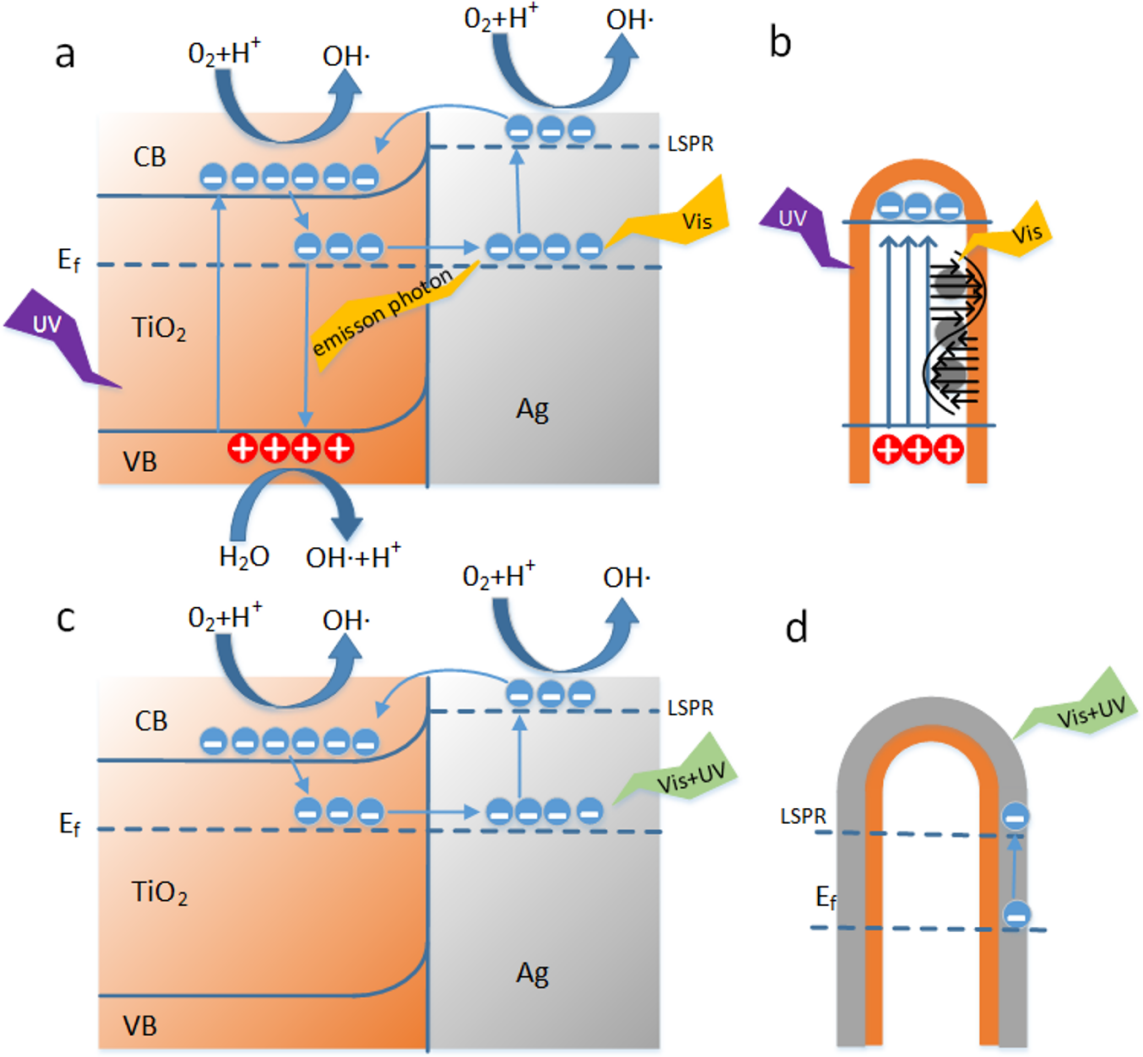
(a) Schematic diagram of electron–hole separation and energy conversion of Ag–TiO_2_ structures under UV–vis illumination. b) AFT structure light absorption diagram. c) Schematic diagram of energy conversion in the case of excessive Ag-coated TiO_2_. d) ACT structure light absorption diagram.

Figure 9a shows the photoelectric energy conversion of the Ag–TiO_2_ structure. The contact between Ag and the n-type semiconductor TiO_2_ will form a Schottky barrier at the interface, which results in the bending of the energy band of TiO_2_ [37]. Due to the influence of SPR and LSPR effects, Ag can absorb visible light and make the electron near the Fermi level transition to a higher level [38]. When the high-level electrons have enough energy, the electrons will cross the Schottky barrier, transfer to the conduction band of TiO_2_, or directly participate in the catalytic reaction [39]. Because of the wide bandgap, TiO_2_ can only absorb UV light. After the valence band electron absorbs the photon energy, it transitions from the valence band to the conduction band, and it takes part in the catalytic reaction with the electrons transferred from Ag. In the recombination process, the conduction band electrons that are not involved in the catalytic reaction can occupy the impurity level near the Fermi level and transfer to Ag to participate in the energy conversion process again. At the same time, the light and heat generated by the recombination process can also be absorbed by Ag, thus increasing the concentration of photogenerated electrons and the rate of catalytic reaction [40]. However, when Ag was covered with TiO_2_, the Ag blocked the absorption of ultraviolet light by TiO_2_, resulting in the absence of photogenerated carriers. Only the energetic electrons produced by Ag could participate in the catalytic reaction, as shown in Figure 9c. The above results show that more photo-generated carriers are generated on the AFT structure, and the good conductivity of Ag promotes the separation of photo-generated carriers, reducing the recombination of carriers [41]. The carriers can oxidize dissolved oxygen, H^+^ and H_2_O to active free radicals, and active free radicals can mineralize pollutants [42], where the chemical reaction is described as follows.

[5] $${\text{TiO}}_{2}+h\nu \to {\text{TiO}}_{2}(e^{-},h^{+})$$

[6] $${\text{H}}_{2}\text{O}+h^{+}\to \text{OH}\cdot +{\text{H}}^{+}$$

[7] $${\text{O}}_{2}+e^{-}\to {\text{O}}_{2}^{-}$$

[8] $${\text{H}}_{2}\text{O}+{\text{H}}^{+}+{\text{O}}_{2}^{-}\to \text{OH}\cdot$$

[9] $$\text{OH}\cdot +\;\text{pollutant}\to {\text{H}}_{2}\text{O}+{\text{CO}}_{2}$$

Therefore, the increase of carrier concentration promotes the generation of active radicals, which increases the catalytic reaction rate and the catalytic efficiency [41,43].

In Figure 9b and 9d, a schematic diagram of the energy conversion of the two different nanocolumn structures is shown. In the AFT structure, the good transmittance of TiO_2_ to visible light ensures that the absorption of visible light by Ag will not be hindered. Conversely, in the ACT structure, Ag will over occupy the active site of TiO_2_ [44], which hinders the absorption of photons by TiO_2_ and the contact with MB molecules [45], affecting the catalytic reaction. In addition, since the chemical transformation of MB molecules occurs at the Ag–TiO_2_ interface, Ag is more easily oxidized, which damages the long-term stability of the Ag–TiO_2_ heterojunctions [46]. Taken together, these factors can explain the difference of catalytic efficiency between AFT and ACT.

## Conclusion

By combining the interaction mechanism between noble metal particles and semiconductor materials using a template method, the effect of different loading positions of Ag particles on the catalytic efficiency of Ag-loaded TiO_2_ nanocolumn (Ag-TNC) arrays has been evaluated. According to the results of the photocatalytic experiments, the presence of Ag particles can improve the catalytic efficiency of the TNC structure, but the increase of the Ag deposition thickness reduces the catalytic efficiency of the Ag-coated TNC structure, evidencing the influence of loading location. The morphological analysis of ACT and AFT revealed that the size and distribution density of Ag particles in the ACT structure increase significantly with the deposition thickness, which weakens the LSPR effect. At the same time, the coating Ag particles hinder the absorption of light by TiO_2_ and the contact with reactants, negatively affecting the catalytic reaction. In contrast, the excessive Ag deposition is limited by the special structure of the AFT samples, in which the absorption of visible light is not hindered. This can also be observed from the simulation results. Therefore, the catalytic efficiency of the AFT structure is not affected by the Ag deposition thickness and can always be maintained at a high level. In summary, the Ag-filled TiO_2_ structure has more relaxed preparation parameters and stable catalytic efficiency than its Ag-coated counterpart, which renders it more applicable as a pollutant treatment material.

## References

[1] Chen X, Mao S S (2007). Chem Rev.

[2] Chen X, Shen S, Guo L, Mao S S (2010). Chem Rev.

[3] Asahi R, Morikawa T, Ohwaki T, Aoki K, Taga Y (2001). Science.

[4] Li M, Wang M, Zhu L, Li Y, Yan Z, Shen Z, Cao X (2018). Appl Catal, B.

[5] Ghosh A, Nayak A K, Pal A (2017). Curr Pollut Rep.

[6] Tong H, Ouyang S, Bi Y, Umezawa N, Oshikiri M, Ye J (2012). Adv Mater (Weinheim, Ger).

[7] Diebold U (2003). Surf Sci Rep.

[8] He H-Y, He Z, Shen Q (2018). J Colloid Interface Sci.

[9] Wang X, Xiang Y, Zhou B, Zhang Y, Wu J, Hu R, Liu L, Song J, Qu J (2019). J Colloid Interface Sci.

[10] Fang W, Xing M, Zhang J (2017). J Photochem Photobiol, C.

[11] Zhukovskii Y F, Piskunov S, Lisovski O, Bocharov D, Evarestov R A (2017). Isr J Chem.

[12] Cheng Z, Zhao S, Han Z, Zhang Y, Zhao X, Kang L (2016). CrystEngComm.

[13] Liu X, Iocozzia J, Wang Y, Cui X, Chen Y, Zhao S, Li Z, Lin Z (2017). Energy Environ Sci.

[14] Kelly K L, Coronado E, Zhao L L, Schatz G C (2003). J Phys Chem B.

[15] Jaramillo-Páez C, Navío J A, Hidalgo M C (2018). J Photochem Photobiol, A.

[16] Sohrabnezhad S, Seifi A (2016). Appl Surf Sci.

[17] Yu J, Dai G, Huang B (2009). J Phys Chem C.

[18] Liu B, Aydil E S (2009). J Am Chem Soc.

[19] Yao Z, Wang C, Li Y, Kim N-Y (2015). Nanoscale Res Lett.

[20] Wang Q, Wang X, Zhang M, Li G, Gao S, Li M, Zhang Y (2016). J Colloid Interface Sci.

[21] Dong J, Ye J, Ariyanti D, Wang Y, Wei S, Gao W (2018). Chemosphere.

[22] Wen L, Wang Z, Mi Y, Xu R, Yu S-H, Lei Y (2015). Small.

[23] Das C, Richter M, Tallarida M, Schmeisser D (2016). J Phys D: Appl Phys.

[24] Govorov A O, Zhang H, Gun’ko Y K (2013). J Phys Chem C.

[25] Li J, Cushing S K, Zheng P, Meng F, Chu D, Wu N (2013). Nat Commun.

[26] Awazu K, Fujimaki M, Rockstuhl C, Tominaga J, Murakami H, Ohki Y, Yoshida N, Watanabe T (2008). J Am Chem Soc.

[27] Wu N (2018). Nanoscale.

[28] Cushing S K, Bristow A D, Wu N (2015). Phys Chem Chem Phys.

[29] Wang X, Wang Z, Jiang X, Tao J, Gong Z, Cheng Y, Zhang M, Yang L, Lv J, He G (2016). J Electrochem Soc.

[30] Sung D, Kim H G, Cha S K, Kim D H, Lee H-B-R, Kim S O, Kim D W, Yeom G Y (2017). Nanosci Nanotechnol Lett.

[31] Jani N A, Haw C Y, Chiu W S, Rahman S A, Lim Y C, Khiew P S, Yaghoubi A (2017). Mater Charact.

[32] Yang J, Jiang Y-L, Li L-J, Muhire E, Gao M-Z (2016). Nanoscale.

[33] Ji N, Ruan W, Li Z, Wang C, Yang Z, Zhao B (2013). J Raman Spectrosc.

[34] Vijayalakshmi R, Rajendran V (2012). Arch Appl Sci Res.

[35] May-Lozano M, Ramos-Reyes G M, López-Medina R, Martínez-Delgadillo S A, Flores-Moreno J, Hernández-Pérez I (2014). Int J Photochem.

[36] Fendrich M A, Quaranta A, Orlandi M, Bettonte M, Miotello A (2019). Appl Sci.

[37] Chuang H-Y, Chen D-H (2011). Int J Hydrogen Energy.

[38] Chen D, Chen Q, Ge L, Yin L, Fan B, Wang H, Lu H, Xu H, Zhang R, Shao G (2013). Appl Surf Sci.

[39] Tan L-L, Ong W-J, Chai S-P, Mohamed A R (2015). Appl Catal, B.

[40] Zhang Z, Yates J T (2012). Chem Rev.

[41] Li J-F, Zhong C-Y, Huang J-R, Chen Y, Wang Z, Liu Z-Q (2019). J Colloid Interface Sci.

[42] Gunti S, Kumar A, Ram M K (2018). Int Mater Rev.

[43] Wetchakun K, Wetchakun N, Sakulsermsuk S (2019). J Ind Eng Chem (Amsterdam, Neth).

[44] Guan H, Wang X, Guo Y, Shao C, Zhang X, Liu Y, Louh R-F (2013). Appl Surf Sci.

[45] Subramanian V, Wolf E, Kamat P V (2001). J Phys Chem B.

[46] Gomathi Devi L, Kavitha R (2016). Appl Surf Sci.

